# The European Researchers’ Network Working on Second Victim (ERNST) Policy Statement on the Second Victim Phenomenon for Increasing Patient Safety

**DOI:** 10.3389/phrs.2024.1607175

**Published:** 2024-09-18

**Authors:** Jose Mira, Irene Carillo, Susanna Tella, Kris Vanhaecht, Massimiliano Panella, Deborah Seys, Marius-Ionut Ungureanu, Paulo Sousa, Sandra C. Buttigieg, Patricia Vella-Bonanno, Georgeta Popovici, Einav Srulovici, Sofia Guerra-Paiva, Bojana Knezevic, Susana Lorenzo, Peter Lachman, Shin Ushiro, Susan D. Scott, Albert Wu, Reinhard Strametz

**Affiliations:** ^1^ Fundación para el Fomento de la Investigación Sanitaria y Biomédica de la Comunitat Valenciana (FISABIO), Valencia, Spain; ^2^ Health Psychology Department, Miguel Hernández University of Elche, Elche, Spain; ^3^ Health Care and Social Services, LAB University of Applied Sciences, Lappeenranta, Finland; ^4^ Leuven Institute for Healthcare Policy, Leuven University, Leuven, Belgium; ^5^ Dipartimento di Medicina Traslazionale, Università del Piemonte Orientale UPO, Novara, Italy; ^6^ Faculty of Political, Administrative and Communication Sciences, Babeș-Bolyai University, Cluj-Napoca, Romania; ^7^ Public Health Research Centre, Comprehensive Health Research Center (CHRC), Lisbon, Portugal; ^8^ NOVA National School of Public Health, NOVA University, Lisbon, Portugal; ^9^ Department of Health Systems Management and Leadership, Faculty of Health Sciences, University of Malta, Msida, Malta; ^10^ Institutul National de Management al Serviciilor de Sanatate Romania, Bucuresti, Romania; ^11^ Department of Nursing, University of Haifa, Haifa, Israel; ^12^ University Hospital Centre Zagreb, Zagreb, Croatia; ^13^ Hospital Universitario Fundación Alcorcón, Alcorcón, Spain; ^14^ Royal College of Physicians of Ireland, Dublin, Ireland; ^15^ Division of Patient Safety, Kyushu University, Fukuoka, Japan; ^16^ Health Care, Columbia, KY, United States; ^17^ Bloomberg School of Public Health, Johns Hopkins University, Baltimore, MD, United States; ^18^ Wiesbaden Business School, RheinMain University of Applied Sciences, Wiesbaden, Germany

**Keywords:** adverse events, patient safety, healthcare workforce, second victim phenomenon, health worker safety

## Abstract

**Background:**

The second victim phenomenon refers to the emotional trauma healthcare professionals experience following adverse events (AEs) in patient care, which can compromise their ability to provide safe care. This issue has significant implications for patient safety, with AEs leading to substantial human and economic costs.

**Analysis:**

Current evidence indicates that AEs often result from systemic failures, profoundly affecting healthcare workers. While patient safety initiatives are in place, the psychological impact on healthcare professionals remains inadequately addressed. The European Researchers’ Network Working on Second Victims (ERNST) emphasizes the need to support these professionals through peer support programs, systemic changes, and a shift toward a just culture in healthcare settings.

**Policy Options:**

Key options include implementing peer support programs, revising the legal framework to decriminalize honest errors, and promoting just culture principles. These initiatives aim to mitigate the second victim phenomenon, enhance patient safety, and reduce healthcare costs.

**Conclusion:**

Addressing the second victim phenomenon is essential for ensuring patient safety. By implementing supportive policies and fostering a just culture, healthcare systems can better manage the repercussions of AEs and support the wellbeing of healthcare professionals.

## Background

Promoting patient safety remains a paramount objective within global healthcare systems. Despite concerted efforts to minimize adverse events (AEs) in both hospital and primary care settings, a substantial number of patients continue to experience harm during the course of their treatment and care [1–3]. Notably, 49% of avoidable AEs result in mild consequences, while 12% lead to severe outcomes, including permanent disability or death.

Within Europe, the economic toll of avoidable AEs is estimated to range between 17 and 38 billion euros annually, coupled with the loss of 1.5 million disability-adjusted life years (DALYs) [4]. A staggering 15% of total hospital expenditures can be directly attributed to AEs [5, 6]. However, the most profound cost of AEs, the human toll, defies quantification. Consequently, the WHO Global Patient Safety Action Plan 2021–2030 has been adopted though acknowledging that substantial progress is still required [7].

Following an AE, immediate attention is directed towards addressing the psychosocial, biological, and physical needs of the patient and their relatives. This involves providing clear, understandable, and truthful information regarding the incident, encouraging their involvement in enhancing care, and offering pathways for fair compensation [8–10]. Recognizing that the majority of adverse events stem from systemic failures, it’s also important to address the emotional impact on healthcare professionals to ensure they are in the best condition to provide safe and effective patient care [11–13].

**FIGURE 1:**
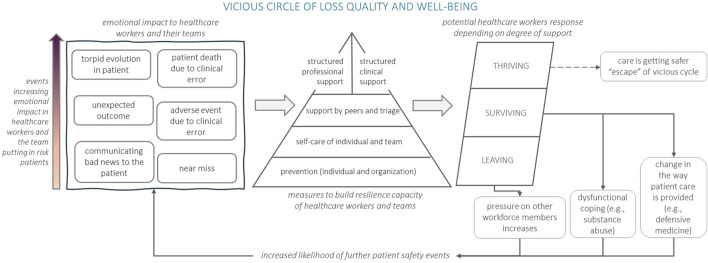
Vicious circle of loss of quality and wellbeing adapted from [15–17] Europe, 2024.

Coined by Albert Wu, the term “second victim” describes what happens to healthcare workers when something goes wrong. They might feel sad, guilty, angry, have flashbacks, feel alone, worry about how patients, colleagues, and their workplace will react, and question their abilities [11]. This distressing experience may progress to disengagement, drop-out, burnout, post-traumatic stress and, in extreme cases, suicide. The ERNST (The European Researchers’ Network Working on Second Victims) Consortium recently refined this definition to encompass: “any healthcare worker, directly or indirectly involved in an unanticipated adverse patient event, unintentional healthcare error, or patient injury, and who becomes victimized in the sense that they are also negatively impacted” [14]. Without proactive measures to restore the mental wellbeing and confidence of healthcare professionals, the psychological toll can compromise their ability to deliver quality and safe care (Figure 1).

Confronting the stigma associated with adverse events (AEs) is crucial for mitigating risks in clinical settings and enhancing overall patient safety. This document presents a concise overview of principles, a conceptual framework, and actionable strategies aimed at diminishing the repercussions of the second victim phenomenon while simultaneously bolstering patient safety.

## Analysis

The ERNST Consortium, funded by the COST Association under action reference CA19113, was officially established on 15 September 2020. Its primary objective is to facilitate an open and comprehensive dialogue among stakeholders concerning the implications of the second victim phenomenon. ERNST serves as a collaborative platform spanning international boundaries, incorporating diverse disciplines and perspectives, including legal, educational, professional, and socio-economic considerations.

A consensus-building process was initiated, coordinated by the leaders of the working groups constituting the Core Group of this COST Action 19113. The engagement of Consortium members representing 29 European countries and 15 inclusiveness target countries was integral to this procedure. This statement, endorsed by the ERNST Management Committee’s leading professionals, research group leaders, and department heads in hospitals and primary care, reflects the outcomes of extensive deliberations, including exchanges of experiences, webinars, workshops, and forums held during this Action. Consultations with experts from Europe and beyond were undertaken, and their insights were considered in reviewing contributions and shaping the final declaration, leading to the formulation of proposed clarifications. All reflections and ideas discussed in the various forums organized by ERNST, along with experiences and recent publications that fueled the debates, were systematically categorized into key components. These themes were used to extract the main points of consensus, which were then formalized into actionable proposals. The ERNST Consortium has articulated this policy statement, organized into five distinct components.

## Policy Options

### ERNST Policy Statement

1. Ensuring patient safety is a global priority.
  1.1. The complexity of healthcare and clinical environments requires healthcare institutions to anticipate, manage and control risks, and respond to AEs with system wide learning [18, 19].
  1.2. Most AEs have a multifactorial and systemic origin. They result from a combination of latent conditions and system failures that can lead to patient harm, which may include clinical error [20].
  1.3. Patient safety is a cross-cutting dimension of quality of care. Healthcare institutions need to have a system mitigating healthcare risks that leads to continuous improvement and the creation of learning organizations.
2. Ensuring healthcare provider capacity is a priority.
  2.1. Healthcare is an emotionally demanding profession. A commitment from the European Commission is necessary, urging countries to establish national programs on occupational health and safety for healthcare workers, in line with WHO recommendations [21].
  2.2. Following any safety incident or unexpected patient outcome, prioritizing patient care is essential. This care must not overlook the psychological impact of the adverse event. The impact on healthcare professionals [22–27], health science trainees and students [28, 29] as second victims must also be addressed to ensure proper patient care. This human reaction occurs in a similar way among informal caregivers at home [30, 31].
  2.3. In situations causing distress and uncertainty, individuals naturally react and question their actions. Without a supportive organizational environment and emotional support, these reactions can have long-lasting negative consequences on patients, the professional team, and individuals themselves [15, 32–37]. In the most severe cases, the second victim’s experience can trigger post-traumatic stress disorder (estimated prevalence ranging from 5% to 17%) [38] or even suicide [39]. Patient safety and quality care plans and programs at local, regional and national levels must not be designed without considering this reality.
3. Allocating resources in second victim support.
  3.1. There is evidence supporting the effectiveness and acceptability of peer support programs with trained supporters [40–44]. These programs should be implemented at local level alongside preventive measures and initiatives that promote emotional self-care and resilience [16], helping healthcare professionals manage the highly stressful situations inherent in clinical practice. Failing to do so puts patient safety at risk [21].
  3.2. Peer support is the most desired, accepted, feasible, and affordable modality of support for healthcare organizations [37, 45–47]. The initial peer support programs began in the US and have been in operation for approximately 14 years [42, 48]. These programs offer emotional assistance to second victims through institutionally-designed initiatives, and in certain instances, encompass a network of hospitals for wider impact. Depending on the country’s organizational models, these programs could be managed by departments of patient safety, occupational health, human resources, or independently. Psychosocial support has been extended to unexpected and tragic events affecting the healthcare professionals, which escalated during the COVID-19 pandemic [49].
  3.3. Moreover, implementing a peer support program results in net monetary savings. Evidence suggests estimated cost savings to single healthcare institutions of 1€ million per year [45, 50]. This cost is considerably increased if we consider the loss of competent healthcare professionals as well as the costs inherent to defensive medicine [51, 52].
4. Re-thinking legal framework and building just culture.
  4.1. The promotion of just culture principles within healthcare organizations is essential [53]. A regulatory change to decriminalize honest clinical errors, as in civil aviation, is essential to shift from a reactive culture to one that fosters safety.
  4.2. The complexity of the second victim phenomenon requires solutions beyond enhancing resilience [16, 54, 55]. It is recommended to advance in the analysis and discussion of alternatives to the traditional legal framework [56]. Leveraging the experiences of countries that have embraced no-fault systems and instituted modifications in claims and compensation procedures can provide valuable insights for progress in this regard [57–60].
5. ERNST Commitment to successful actions
  5.1. Increase awareness of all stakeholders (European, national and regional levels) to facilitate discussion of the legal, ethical, social, and organizational issues deterring tackle with the impact of the second victim phenomenon in patient safety.
  5.2. Examine more thoroughly the consequences of medication and care errors among informal caregivers of dependent patients at home, and advocate for local-level initiatives to mitigate their effects.
  5.3. The phenomenon of the second victim should not be understood as a problem that falls exclusively on healthcare organizations, their professionals, and patients, as its effective management involves society as a whole. This issue must be addressed by health systems, healthcare institutions, and the organizations representing professionals, patients and citizens. Health authorities and policymakers at the national as well as at the international level should consider these aspects and act in accordance with the scientific evidence.

## Conclusion

Drawing on international collaboration and consultations, the statement emphasizes the need for comprehensive approaches, peer support programs, and re-evaluation of legal frameworks. By fostering dialogue among stakeholders and advocating for systemic changes, this policy statement aims to cultivate a supportive environment for healthcare workers and ultimately improve the quality and safety of patient care.

## Group Member of ERNST Consortium

Yolanda Agra Varela, Ministry of Health, Social Services and Equality, Spain; Veli-Jukka Anttila, Helsinki University Central Hospital, Finland; Jesús Aranaz Andrés, Hospital Ramón y Cajal, Spain; Pinar Ayvat, Izmir Democracy University School of Medicine, Türkiye; Elena Bohomol, Universidade Federal de São Paulo, Brazil; Patricia Vella Bonanno, University of Malta, Malta; Gunnar Tschudi Bondevik, University of Bergen, Norway; Helga Bragadottir, University of Iceland, Iceland; Sandra C. Buttigieg, University of Malta, Malta; Irene Carrillo, Miguel Hernández University of Elche, Spain; Mery González Delgado, Iberoamerican Network of Knowledge in Patient Safety and Nursing LATAM, Colombia; Ezequiel García-Elorrio, Institute for Clinical Effectiveness and Health Policy, Buenos Aires, Argentina; Brigitte Ettl, Wiener Gesundheitsverbund, Austria; Aikaterini Flora, Simple Live POS, Greece; Anatolii Goncharuk, International Humanitarian University, Ukraine; Sofia Guerra, National School of Public Health Universidade NOVA de Lisboa, Portugal; Bojana Knežević, University Hospital Centre Zagreb, Croatia; Hana Knežević Krajina, Health Center Zagreb-Centar, Croatia; Peter Lachman, Royal College of Physicians of Ireland, Ireland; Susana Lorenzo, Hospital Universitario Fundación Alcorcón, Spain; Neda Milevska Kostova, Institute for Social Innovation, North Macedonia; Erika Kubiliene, Vilniaus kolegija/Higher Education Institution, Lithuania; Halina Kutaj-Wasikowska, Centrum Monitorowania Jakości, Poland; Barbara Kutryba, Towarzystwo Promocji Jakosci Opieki Zdrowotnej w Polsce (Polish Society for Quality in Healthcare Promotion in Poland) TPJ, Poland; Veronica Lindström, Sophiahemmet Högskola, Sweden; Andrea Madarasova Geckova, Comenius University, FSEV, Institute of Applied Psychology, Slovakia; Jimmy Martín, Hospital Luis Vernaza, Junta de Beneficencia de Guayaquil, Guayaquil, Ecuador; Philippe Michel, Université Lyon 1, France; José Joaquín Mira, Foundation for the Promotion of Health and Biomedical Research of Valencia Region, FISABIO, Spain; Marina Odalovic, University of Belgrade - Faculty of Pharmacy, Serbia; Massimiliano Panella, Università del Piemonte Orientale UPO, Italy; Rodrigo Poblete, Universidad Católica de Santiago de Chile, Chile; Kaja Põlluste, University of Tartu, Estonia; Georgeta Popovici, Institutul National de Management al Serviciilor de Sanatate Romania, Romania; Anat Rafaeli, Technion - Israel Institute of Technology, Israel; David Schwappach, Swiss Patient Safety Foundation, Switzerland; Silvia Gabriela Scintee, Institutul National de Management al Serviciilor de Sanatate, Romania; Susan D. Scott, University of Missouri, United States; Piedad Serpa, Universidad de Santander, Bucaramanga, Colombia; Deborah Seys, KU Leuven–University of Leuven, Belgium; Raluca Sfetcu, Institutul National de Management al Serviciilor de Sanatate Romania, Romania; Sigurbjorg Sigurgeirsdottir, University of Iceland, Iceland; Ivana Skoumalova, Pavol Jozef Safarik University, Slovakia; Paulo Sousa, National School of Public Health Universidade NOVA de Lisboa, Portugal; Einav Srulovici, University of Haifa, Israel; Nebojša Stilinović, University of Novi Sad, Faculty of Medicine Novi Sad, Serbia; Reinhard Strametz, RheinMain University of Applied Sciences, Germany; Savka Strbac, Public Health Institute of the Republic of Srpska, Bosnia and Herzegovina; Susanna Tella, LAB University of Applied Sciences, Finland; Viktoriia Tkachenko, Granddent, Ukraine; Mary Tumelty, University College Cork, Ireland; Marius-Ionut Ungureanu, College of Political, Administrative and Communication Sciences, Romania; Shin Ushiro, Kyushu University, Japan; Kris Vanhaecht, KU Leuven–University of Leuven, Belgium; Albert W. Wu, Johns Hopkins Bloomberg School of Public Health, United States.
